# Hydatid Cyst in the Lumbar Paravertebral Muscle: A Case Report

**DOI:** 10.7759/cureus.5353

**Published:** 2019-08-09

**Authors:** Homa Sadeghian, Rouzbeh Motiei-Langroudi

**Affiliations:** 1 Surgery, University of Pittsburg Medical Center Pinnacle, Harrisburg, USA; 2 Neurosurgery, University of Kentucky, Lexington, USA

**Keywords:** hydatid cyst, paravertebral muscle, multifidus muscle, resection

## Abstract

A hydatid cyst is a zoonotic disease caused by the worm Echinococcus granulosus. In endemic regions, it is a well-known differential diagnosis of cystic lesions, especially in the liver, lungs, brain, and vertebral column. Primary paravertebral muscle involvement, however, is rarely reported. In the current report, we present the case of an 11-year-old girl complaining of back pain with a well-defined single cystic lesion in her lumbar paravertebral multifidus muscle evident in imaging studies. The patient had no concomitant lesion in her systemic evaluation. The cyst was resected totally with its daughter cysts, and the pathology confirmed the diagnosis of a hydatid cyst. Although the paravertebral muscle is an extremely rare site of infection for a hydatid cyst, it should be kept in mind in mass lesions with a cystic nature.

## Introduction

A hydatid cyst or hydatidosis is a zoonotic disease with a worldwide distribution. The disease may be responsible for approximately 1% of surgeries for mass lesions in endemic regions [1-3]. Dogs infected with E. granulosus or consummation of contaminated water or vegetables are widely considered as the main source of infection for hydatid cysts in humans [4].

It most commonly affects the liver and lungs, as the parasites penetrate the duodenal mucosa, enter the portal circulation, and infect the liver and lung first. The parasites less frequently enter the systemic circulation and disseminate throughout the body to infect other organs, including the brain, vertebral column, and spinal cord [5-6]. Musculoskeletal involvement is rare and contributes to 0.5%-5% of cases of hydatid cysts and is almost always secondary to liver or lung infection [2]. Primary musculoskeletal and, in particular, muscular involvement without visceral infection is extremely rare [7]; therefore, it may be a difficult and challenging differential diagnosis. Among musculoskeletal sites, there are only a few cases reporting the primary involvement of paravertebral muscles [2,5,8-10].

In the current report, we present a case of a single hydatid cyst involving the paravertebral multifidus muscle in a young girl complaining of back pain.

## Case presentation

**Figure 1:**
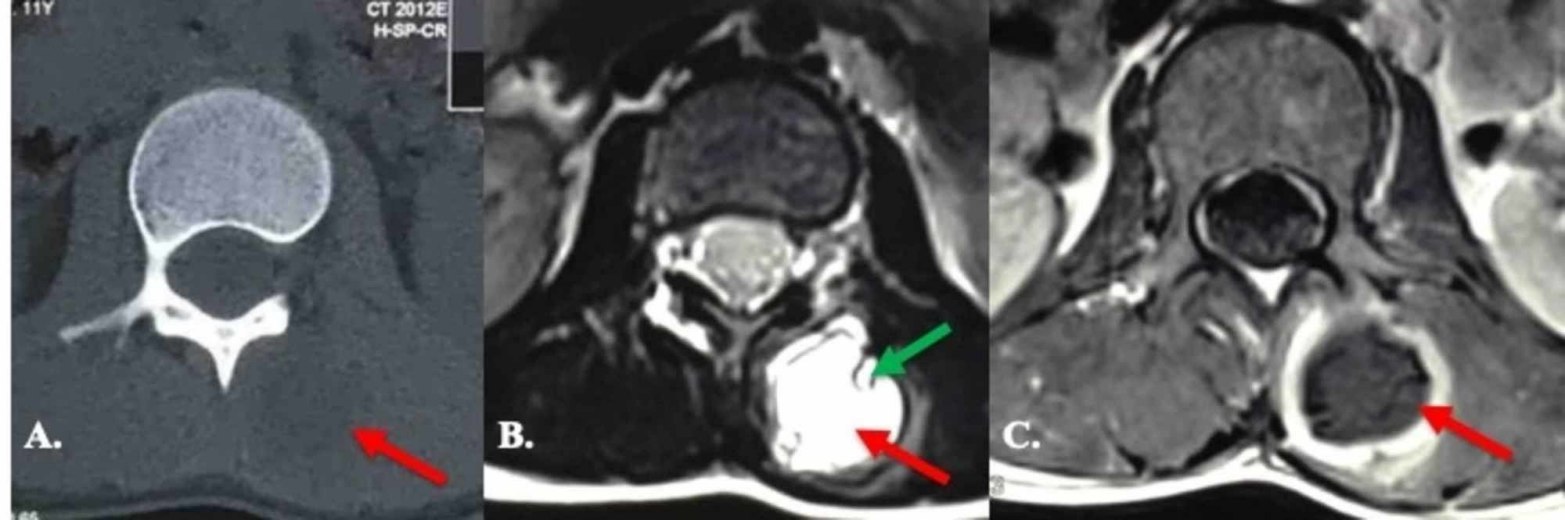
Imaging A) CT scan; B) T2-weighted MRI; C) Contrast-enhanced T1 MRI of the lesion. The red arrow shows the cyst itself while the green arrow shows a daughter cyst.

The patient was an 11-year-old girl presenting with back pain; the pain had started two months before presenting to a referral academic neurosurgery clinic in South Iran (Bam University of Medical Sciences Clinic, Bam, Iran). The back pain was localized in the left upper lumbar area. The general examination (lung, liver, lymph nodes, etc) was normal. The neurologic examination (including muscle strength, sensory examination, deep tendon reflexes, etc.) was also normal. On examination, there was a two-by-two centimeter (cm) swelling without tenderness, erythema or rubor in the left upper lumbar paravertebral area (approximately two cm from midline). Laboratory tests remained within the normal range. Chest X-ray (XR), anteroposterior (AP) and lateral abdominal XRs, abdominal ultrasonography, and chest and abdominal computed tomography (CT) couldn't detect any pathology, except a hypodense cystic mass in the left paravertebral muscle without any evidence of bony erosion. Magnetic resonance imaging (MRI) showed a well-defined, capsulated, three-by-three cm cystic mass, hypointense in T1 and hyperintense in T2 with multiple intracystic loculations within the multifidus muscle boundaries from L1 to L3. The mass had peripheral enhancement after Gadolinium injection. The mass showed no involvement of the spinal column, cord, or epidural and subdural spaces (Figure 1).

**Figure 2:**
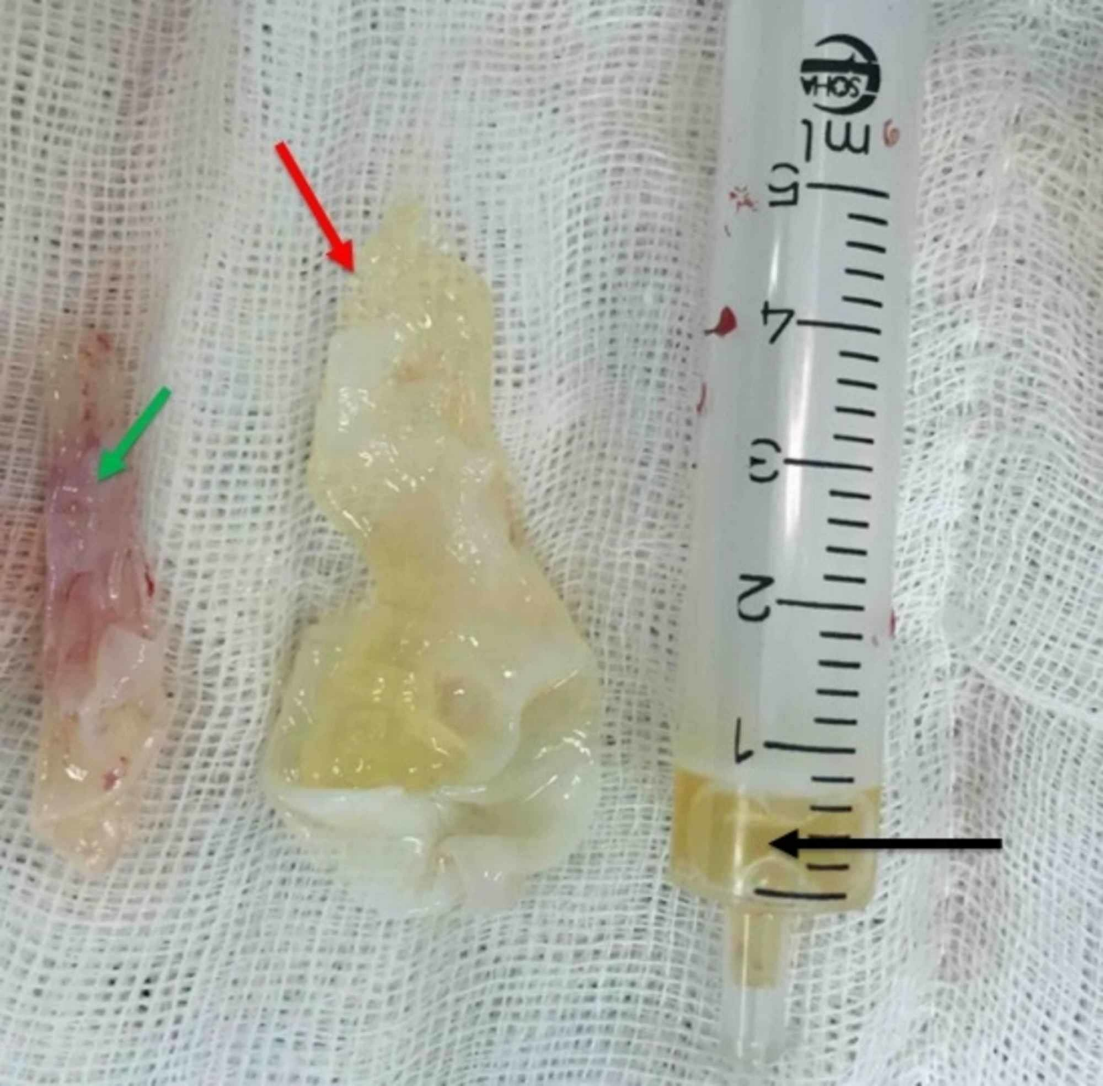
The cyst The mother lesion (red arrow) with a daughter cyst (green arrow) after surgical resection. The syringe (black arrow) is partially filled with the cyst fluid.

The patient underwent surgery in the prone position. A vertical three-cm incision was made over the palpated mass. Subcutaneous tissue, muscle fascia, and a multifidus muscular layer over the mass were sharply dissected. A well-defined, white, pearl-like semi-translucent cyst with adhesion to the surrounding muscle was seen. The cyst was filled with yellow clear fluid and numerous smaller cysts (“daughter cysts”) with a thin white layering were observed. The mother and all daughter cysts were resected along with a very thin layer of surrounding muscle (Figure 2).

No calcification was observed. The vertebral facets and lamina were visible and palpable, with no bony involvement and erosion. The operative field was irrigated with sterile saline and alcohol, and the surgical wound was closed in multiple layers. The whole procedure was uneventful and the patient was discharged the following day.

The pathologic examination by two different pathologists blind to the results of each other confirmed the diagnosis of hydatid cyst. The patient was treated by the administration of albendazole for a total duration of six months after the surgery. On follow-up one year after the surgery, the patient was asymptomatic, without clinical and imaging recurrence.

The patient and her family had given their consent for all diagnostic and therapeutic (surgery and further medications) procedures, as well as the publication of the report.

## Discussion

A hydatid cyst is a zoonotic disease caused by the helminth Echinococcus granulosus. It has a worldwide distribution and is endemic in specific regions; among them are Mediterranean countries and the Middle East [2-3]. It continues its life cycle in dogs and sheep and infects humans with excessive exposure to these animals or through the ingestion of food and water contaminated by their feces [4].

The most common site of infection is the liver and lungs; after distribution in the systemic circulation, every other organ can also be infected. Therefore, in endemic countries, a hydatid cyst should be considered as a differential diagnosis of mass lesions that have a cystic nature. However, even in endemic countries, it rarely affects musculoskeletal and soft tissues and, almost always, this site of infection is accompanied by a liver or lung infection [2,7], as these organs act as the port of entry of parasites after penetrating the duodenal wall [5-6].

Our case was an 11-year-old girl, without a history of recurrent exposure to dogs or sheep, who presented with a history of back pain without radiation to the lower extremities. The pain was accompanied by a non-tender paravertebral swelling. The CT was non-diagnostic, as it only showed a hypodensity within paravertebral muscles. MRI, however, was more helpful, showing multiple loculations and septations within the capsulated cyst and a ring enhancement, suggesting an active hydatid cyst as well as abscess formation as the possible diagnoses. In our case, albeit, MRI failed to show the characteristic “water lily” sign seen in hydatid cysts [10]. Evaluation for concomitant lesions was negative in our patient. The cyst was totally and uneventfully resected in the patient, as en bloc resection without rupture of the mother cyst is considered the treatment of choice for intramuscular hydatid cysts [1].

The sole and primary involvement of a lumbar paravertebral muscle without concomitant liver or lung involvement has been just reported in a few case reports [2,5,8-10]. Therefore, diagnosis may be challenging in such cases given that serologic tests may be negative in isolated muscular hydatid cysts [11]. In most reports, the patient had multiple cysts diagnosed before surgery while our case had a single cyst within her multifidus paravertebral muscle, adding to the difficult preoperative diagnosis. However, correct diagnosis before surgery is of utmost importance. In this case, for instance, any attempt to obtain a needle biopsy or aspirate the cyst before surgery would only cause dissemination of the parasite and involvement of other anatomical areas. Therefore, in endemic areas, any cystic lesion should raise suspicion for this disease, especially if associated with the classic imaging findings.

## Conclusions

In the current report, we presented a patient with a solitary hydatid cyst in the paravertebral multifidus muscle, even though the disease is known more to involve the lungs, liver, or other organs. The cyst was successfully removed surgically, followed by medical treatment for the infection. Although the paravertebral muscle is an extremely rare site of involvement for hydatid cysts, they should be suspected in patients with cystic lesions who have lived or traveled to endemic areas. A high level of suspicion is needed to plan for the best treatment protocol to avoid preoperative or intraoperative cyst rupture.
